# Epstein-Barr Virus as a Promising Immunotherapeutic Target for Nasopharyngeal Carcinoma Treatment

**DOI:** 10.1155/2017/7349268

**Published:** 2017-12-31

**Authors:** Sin-Yeang Teow, Hooi-Yeen Yap, Suat-Cheng Peh

**Affiliations:** ^1^Sunway Institute for Healthcare Development (SIHD), Sunway University, Jalan Universiti, Bandar Sunway, 47500 Subang Jaya, Selangor Darul Ehsan, Malaysia; ^2^Anatomical Pathology Department, Sunway Medical Centre, Jalan Lagoon Selatan, Bandar Sunway, 47500 Subang Jaya, Selangor Darul Ehsan, Malaysia

## Abstract

Epstein-Barr virus (EBV) is a pathogen that infects more than 90% of global human population. EBV primarily targets B-lymphocytes and epithelial cells while some of them infect monocyte/macrophage, T-lymphocytes, and dendritic cells (DCs). EBV infection does not cause death by itself but the infection has been persistently associated with certain type of cancers such as nasopharyngeal carcinoma (NPC), Burkitt's lymphoma (BL), and Hodgkin's lymphoma (HL). Recent findings have shown promise on targeting EBV proteins for cancer therapy by immunotherapeutic approach. Some studies have also shown the success of adopting EBV-based therapeutic vaccines for the prevention of EBV-associated cancer particularly on NPC. In-depth investigations are in progress to refine the current therapeutic and vaccination strategies. In present review, we discuss the highly potential EBV targets for NPC immunotherapy and therapeutic vaccine development as well as addressing the underlying challenges in the process of bringing the therapy and vaccination from the bench to bedside.

## 1. Introduction

Cancer is one of the top diseases that causes major global death. It is estimated that EBV-associated cancers account for approximately 1.5% of all cancers worldwide and are responsible for 1.8% cancer-related deaths [1]. EBV is closely linked to various type of cancers [2] and has been a promising target for cancer diagnostics, therapy, and vaccine development [3]. EBV largely contributes to NPC, BL, HL, and non-HL while a small percentage of breast cancer, gastric carcinoma (GC), and cervical cancer are also thought to be attributed to EBV infection [4]. EBV can establish either lytic or latent phase in the target cells and both phases contribute differently to cancer development and progression [5]. Almost all of NPC cases are EBV-associated, and the viruses are predominantly latent phase. NPC expresses type III latent genes such as EBV noncoding RNAs (EBER), EBV nuclear antigen 1 (EBNA1), latent membrane proteins (LMPs), and EBV Bam H1-A region rightward transcripts (BARTs). Latent-related proteins such as LMP1 are expressed in almost all NPC tissues [6] while LMP2 is detected in approximately 50% of primary NPC tissues [7]. Both of these oncoproteins have been known to play pivotal roles in carcinogenesis [8]. Similarly, lytic phase-controlling genes such as transcription activator (*BZLF1* and* BRLF1*) and* BMRF1* are also readily detected in NPC [9]. Interestingly, recent articles have highlighted the role of EBV lytic reactivation in NPC including promoting genome instability, invasiveness, and tumorigenesis [10] as well as enhancing secretion of protumorigenic growth and angiogenic factors [11]. To date, the coexistence of EBV latent and lytic phases is mainly reported in NPC [12]. In other EBV-related malignancies the viral infection is latent.

Due to the pathogenic role of EBV in cancer development, focus has been drawn on targeting EBV for cancer therapy in recent years [13, 14]. For example, LMP-specific autologous cytotoxic T-lymphocytes (CTLs) therapy has been an effective treatment in recurrent NPC patients [15]. Interestingly, LMP1-based therapeutic vaccine also suppressed tumour growth and metastasis in mouse models [16]. EBV-based vaccines for cancer control in humans have also been developed. Clinical trials are ongoing to evaluate their uses in NPC patients as therapeutic vaccines after the primary treatment to prevent recurrence [17]. They are not used as prophylactic vaccine for disease prevention. To date, the prophylactic vaccine has only been focused on infectious mononucleosis (IM) targeting EBV gp350 rather than EBV-related malignancies [3, 18]. EBV proteins that are currently being targeted for therapeutic vaccine development are mainly LMP2A and EBNA1 [19]. From a Phase 1A trial on NPC patients in Hong Kong and UK, the modified vaccinia Ankara- (MVA-) based LMP2 and EBNA1 (MVA-LMP2/EBNA1) vaccine has resulted in a postvaccination immune boosting of CD8+ and CD4+ T-cell responses with low off-target toxicities in both Chinese and European descents [19, 20].

Collective findings showed that immunotherapy or vaccine development against EBV proteins (particularly LMP1, LMP2A, and EBNA1) for cancer therapy is promising. Albeit EBV has been studied for many years, there is still a big gap of our understanding on its exact pathogenic role in cancers [2]. In the near future, it is expected that the increasing knowledge on EBV as risk factor and biomarker for EBV-associated cancers will contribute to early diagnosis and prediction in treatment outcomes [14]. In this review, we discuss the EBV-targeting immunotherapy and EBV-derived vaccines on NPC as well as delineating the potential challenges in developing them into clinics and possible ways to circumvent these problems.

## 2. Contribution of EBV Proteins to NPC

NPC is closely associated with EBV; the viral proteins are believed to play important roles in augmenting the cancer development and progression. This has been recently discussed in several reviews [2, 38].  Table 1 summarizes the probable pathogenic roles of numerous EBV proteins in NPC including both latent and lytic proteins. Amongst all the EBV proteins, LMPs, the latent proteins, are the key determinants for NPC pathogenesis. LMP1 and LMP2 proteins are readily detected in primary NPC tissues [14]. Both LMPs are oncoproteins that activate and transform the infected cells and enhance the cell proliferation and survival [2, 5]. Generally, LMPs have poor immunogenicity; however LMP2 proteins are relatively more immunogenic than LMP1, hence serving as a more important target for EBV-directed immunotherapy [39]. Notably, it has been reported that high expression of LMP1 (rarely reported in NPC) may inhibit epithelial cell growth and induce apoptosis instead of promoting cancer growth [6]. Another EBV latent protein, EBNA1, is also consistently detected in NPC tissues. The main functions of this protein are to maintain the viral DNA in cells during division, modulate both viral and host genes, and regulate the related cellular pathways in EBV-associated cancers [40]. EBNA1 is a dominant target for CD4+ T-cells and can be detected by CD8+ T-cells upon induction via cross-presentation by professional antigen presentation cells (APC) [41, 42]. The pathogenic role of EBNA1 in NPC is summarized in Table 1.

In the past five years, research strongly suggests that NPC development is attributed to lytic reactivation of EBV which resulted in the reversed viral tropism from lymphotropic to epitheliotropic [12]. This lytic EBV strain was first reported by Tsai et al., and the NPC-derived strain was designated as M81 [43]. This virus was derived from a Chinese NPC patient and has shown high expression level of lytic genes such as* BZLF1* and* BALF* in the M81-infected cells [43]. Further investigations on M81 have been carried out since the first study [44, 45]. However, there is only one EBV strain with these unique characteristics in the world to date. The roles of several EBV lytic proteins in NPC are summarized in Table 1. Although EBV lytic genes may contribute to NPC pathogenesis, activating the lytic cycle of EBV by various compounds have been shown to suppress NPC growth which can be an alternative strategy for NPC therapy [13, 46–48]. In a NPC-directed EBV-specific immunotherapy, Louis et al. demonstrated that the CTLs were reactive against three lytic antigens (Zta, Rta, and BMLF1) in addition to other latent proteins [49]. This study again suggested the potential of targeting lytic proteins for treating NPC. Further studies are required to closely link up the EBV lytic reactivation with NPC establishment. All in all, the M81 strain highlighted that the lytic genes or proteins may also be the important determinants in (a) enhancing epithelial cell infection; (b) EBV cell-to-cell spreading; and (c) early detection marker for EBV cancers. Whether these lytic proteins can be potential target for immunotherapies or vaccine development deserves further investigations.

## 3. Targeting EBV for NPC Immunotherapy and Vaccines

Radiation and/or chemotherapy are standard therapies for NPC, but their side effects are notorious. This urges the need of developing a safe, well-tolerant, and effective treatment. Other than drug-based treatment, EBV-specific CTL immunotherapy has shown promise in treating NPC [14, 50]. In addition, EBV-derived therapeutic vaccine can also be developed and used as adjuvant therapy to prevent NPC recurrence [14, 17].  Table 2 summarizes the evidences of prominent EBV-specific immunotherapy and vaccine development against NPC in the past 10 years. Thus far, the highly targeted EBV proteins for both therapy and vaccine development are LMP1, LMP2, and EBNA1.

In the development of EBV-specific immunotherapy against NPC, autologous cells mainly CTL [15, 53] and dendritic cells (DCs) [12, 56, 57] are expanded* ex vivo* and characterized before infusing into the NPC patients. The characterization of the CTLs usually include sterility, immunophenotype (e.g., T-cell and or natural killer cell population), EBV-specificity, and human leukocyte antigen- (HLA-) type [49, 54]. In most if not all studies, the expanded CTLs largely comprise CD3+ T-cells (>80%) (mostly CD8+ T-cells) followed by natural killer (NK) cells [49, 54]. The patients who receive the infusion are closely monitored to evaluate the safety, tolerance, and efficacy of the therapy. The clinical responses of the patients are often assessed by analysis of EBV DNA and cytokine secretion from the plasma or serum together with the clinical examination (i.e., tumour relapse, regression, or recurrence). This clinical information is often correlated with each other to determine the overall clinical benefits of the therapy. Some of the promising EBV-specific immunotherapies against NPC are shown in Table 2.

While acting as promising targets for immunotherapy, EBV proteins have also been developed into therapeutic vaccines. It is important to note that this vaccine is different from those prophylactic vaccines that could prevent viral diseases such as measles-mumps-rubella (MMR) and rabies. This EBV-based vaccination functions by boosting the production of EBV-specific T-cells and the clinical responses rather than generating the antigen-specific antibodies for protection. In the past 10 years, promising data have been shown from two Phase 1A trials conducted in Hong Kong and United Kingdom on NPC patients [20]. The MVA-EBNA1/LMP2 vaccine resulted in a postvaccination increase of LMP2- and EBNA1-specific CD8+ and CD4+ T-cell responses in both Chinese and European descent which have circumvented several limitations including poor immunogenicity, HLA variation, and EBV strain difference [17, 19]. On the other hand, Chia and colleagues demonstrated that adenovirus-ΔLMP1-LMP2-loaded DC vaccine showed clinical responses in 3 of 12 metastatic NPC patients, and the remaining patients demonstrated delayed type hypersensitivity responses, but not the increase of EBV-specific T-cells [56]. These findings are tabulated in Table 2. There are several advantages of EBV vaccination over CTL therapy: (a) it can be produced in large quantity and lower cost; (b) it does not require highly trained staff and facilities; (c) it does not require live host system; and (d) it is highly consistent and reproducible.

The toxic effects have always been a major concern for cancer immunotherapies [58, 59]. The side effects of both EBV-based CTL immunotherapy and therapeutic vaccine are summarized in Table 2. Common mild toxic effects such as flu-like symptoms, fever, fatigue, skin rash, headache, dizziness, nausea, malaise, weakness, abdominal pain, hypotension, haemoptysis, epistaxis, arthralgia, and myalgia [15, 19, 20, 49, 52, 54–56]. Other negative effects were neutropenia [49, 52], thrombocytopenia [52], and anaemia [52, 56]. Immunotherapies or vaccine trials that resulted in grade 3 side effects [53, 55, 56] must be relooked and improved in terms of safety before being used in clinical setting. In a vaccine trial, Taylor and colleagues also pointed out that 9 of the patients who received EBV vaccine had systemic toxicity [19].

## 4. Challenges and Future Perspectives

Cumulative findings suggest that latent proteins, that is, LMP2A and EBNA1, are promising EBV targets for therapeutic vaccine development against NPC. Development of EBV-based prophylactic vaccine in human is almost impossible due to the time taken for NPC establishment and the possibility of developing other EBV-associated malignancies such as BL and HL in the host. As EBV lytic reactivation has been associated with NPC oncogenesis in recent years, further investigations are warranted to evaluate its therapeutic or preventive potential by targeting the lytic genes/proteins, that is, Zta, BALF3, and BARF1. Interestingly, EBV latent infection can also be activated into lytic phase which subsequently caused cell death and tumour suppression in NPC [46–48]. Although EBV and its pathogenesis in cancers have been studied for many years, developing EBV-based immunotherapy or vaccines is challenging. This section aims to delineate the underlying limitations in order to refine the future therapeutic and preventive strategies against NPC.

As the EBV-based therapeutic vaccine development requires the administration of an attenuated full or partial pathogen into the host system, safety is the key issue that must be addressed. While boosting the mucosal immunity, the administration of MVA tuberculosis vaccine is known to be safe without causing any marked side effects [60]. The same vector has been used to construct the therapeutic vaccine MVA-EBNA1/LMP2 [19]. In a Phase IA trials of MVA-EBNA1/LMP2 on NPC patients in Hong Kong and United Kingdom, the vaccine has been proven safe and well-tolerated [20]. On the other hand, the use of adenoviral vector-based vaccine termed AdE1-LMPpoly [54] and Ad-ΔLMP1-LMP2 [56] in the clinical studies did not cause significant adverse effects in the patients. Similarly, EBV-specific CTL therapies have not shown notable toxicities. Secondino and colleagues have shown that the CTL therapy successfully treated the NPC patients without causing any severe adverse events [52]. Lutzky and coworkers also demonstrated that NPC treatment using CTLs targeting EBV LMP causes neither immediate nor long-term toxicity [15]. Generally speaking, the EBV-specific immunotherapy and vaccination on NPC are safe [50, 53]. However, the safety studies must be rationally designed for a longer period of time as the young NPC patients may suffer from the toxicities much later in their lives. These prolonged studies will also benefit Southeast Asia countries with growing elderly or ageing population where NPC is prevalent.

Before entering human trials,* in vivo* testing using animal models is the gold standard. This is substantial for new immunotherapy testing and novel vaccine development to examine injection dosage, tissue biodistribution, therapeutic efficacy, and potential adverse effects following administration [61]. Choice of animal model is extremely important. There have been numerous patient-derived xenograft (PDX) mouse models used for NPC research such as C15, C17, and C18 [62], Xeno-B110 [63], Xeno-284 [63, 64], and an orthotopic model [65]. However, these immunodeficient mouse models (e.g., Swiss nude and NOD-scid gamma or NSG) for cancer biology studies are not suitable for immunotherapy- or vaccine-related work due to impaired immune system. The lack of a suitable animal model remains the major challenge. In the past, a few mouse models have been chosen and used for immunotherapy and/or vaccine development against EBV-associated cancers. For examples, Fu and colleagues demonstrated that EBNA1 peptide-loaded DCs vaccine elicited CD4+ T-cells responses and tumour growth inhibition using several EBNA1-expressing BL mouse models including wild-type B6, CD8-deficient, and MHC class I-deficient mice [66]. Recently, Lin and coworkers successfully suppressed LMP1-enhanced NPC tumour growth and metastasis by injecting LMP1 vaccine into C57BL6/J mice [16]. Furthermore, the LMP1 vaccine was able to prevent LMP1-expressing tumour development when it was given before tumour challenge [16]. Notably, the outcome of animal model is important to test out the feasibility of new immunotherapy or vaccine, but it must not be related to any of clinical trial outcomes due to the biological variations across different models. Furthermore, the fact that xenograft tumours may not fully recapitulate the original characteristics of patients' tumours must be kept in mind.

Conventional treatment such as chemo- and/or radiotherapy remains the standard for treating advanced stage NPC. However, these approaches greatly reduce the patient life quality; hence other alternatives are urgently needed. Since the use of autologous CTL has been proven safe and highly tolerant, increasing demand has been focused on the efficacy of the immunotherapy. The EBV-targeting CTL therapy has successfully treated the 6 of 11 NPC patients where conventional treatment has failed [52]. Smith and coworkers also showed the success of adoptive immunotherapy using* in vitro*-expanded T-cell on 29 NPC patients who were previously treated by chemotherapy [53]. More promisingly, the combination of EBV-specific CTL therapy with first-line chemotherapy [55] and concurrent chemoradiotherapy [67] have also significantly improved the survival outcome in the NPC patients. Further works are warranted to assess the possibility of combining such immunotherapy with other potent NPC treatments such as programmed cell death protein-1 (PD-1) [68] and programmed cell death ligand-1 (PDL-1) inhibitors [69]. It has also been proposed that the combination of immunotherapy and therapeutic vaccine could further enhance the clinical response of NPC patients [17].

Current literature suggests that latent proteins such as LMP1, LMP2, and EBNA1 are ideal targets for immunotherapy and therapeutic vaccine (Table 2). Several studies have also evidenced that lytic proteins such as BZLF1, BRLF1, and BRMF1 could be potential therapeutic targets but further investigations and validations are warranted [49, 55, 70]. It is known that all EBV-associated malignancies including NPC mostly consist of latently infected cells that have high expression of latent proteins types [71]. This makes them ideal target for therapeutics/therapy development. As latent infection usually allows EBV to evade the host's immune response to remain active in the system, the administration of immunotherapy and therapeutic vaccine targeting latent proteins will boost the immune system to redirect the host to kill the EBV-related tumour through immunological processes. In view of the pathogenic roles of EBV proteins in NPC, some studies have shown their potentials as diagnostic markers [14, 71]. For instance, Houali and colleagues showed that EBV LMP1 and BARF1 proteins are present in serum and saliva of NPC patients and can potentially be developed into diagnostic markers [72]. There are fewer studies denoting the potential of using lytic proteins as NPC diagnostic markers [14, 70]. Comparatively, EBV DNA is a more extensively studied diagnostic marker for NPC as recently highlighted by Chan and colleagues [73].

In addition to EBV latent and lytic proteins (Table 1), other EBV-derived RNAs and DNAs such as EBERs, BARTs, and microRNAs (miRNAs) can also potentially serve as therapeutic targets due to their indispensable roles in NPC pathogenesis [14, 73]. However, more studies are warranted to validate this speculation. Another emerging therapeutic target is the EBV-containing exosomes that can be used as biomarkers for EBV cancers [4, 74]; whether these pathogenic vesicles can be targeted for EBV immunotherapy remains unexplored. Precision oncology has become a hot topic in recent years. While EBV is believed to be the causative pathogen for various lympho- and epithelial malignancies, the attention has been drawn on how to precisely diagnose and treat the EBV cancer with a more accurate prediction. This urges the development of robust and sensitive diagnostic and predictive biomarkers for EBV-related cancers, including NPC.

This is an exciting era to treat or prevent cancers by targeting the cancer-causing pathogens. It has been reported by 2014 AACR Cancer Progress Report 2014 that cancers can be prevented by targeting various factors such as tobacco use (33%), obesity (20%), and tumourigenic pathogens (16%) [75]. For EBV-associated cancers, the pathogen-targeting measures for both therapeutic and vaccine development are just beginning to emerge and endeavours will be made in the coming future to address these questions. First, current therapeutic and preventive strategies are only limited to LMP2A, LMP1, and EBNA1 due to the lack of suitable animal model, poor immunogenicity, and adjuvant [18]. Second, current findings showed that dietary, smoking, host HLA, and coinfection by human papilloma virus (HPV) have implications on NPC patients [76, 77]; whether these key factors will significantly affect the outcomes of EBV-based therapy or vaccine remains to be seen. Third, increasing evidences also highlighted the importance of screening EBV lytic proteins and EBV DNA in the early diagnosis of NPC [18]. Further works are required to examine the effect of new EBV-based therapeutic strategy on the changes of this EBV profiling in order to match the early-stage NPC screening program.

## 5. Conclusions

Cumulative findings suggest that EBV is a pathogen that can be targeted for the management of EBV-related cancers, not only on the therapeutic but on preventive measures. LMP2A and EBNA1 are by far the most promising proteins that are used for therapeutic vaccine development; others remain underexplored. The EBV-based application in cancer diagnostics and therapy is expected to expand more rapidly in near future following our increased understandings of EBV's role in carcinogenesis and the therapeutic implication. Surely, identifying the challenges and promptly addressing them will expedite the EBV-based therapeutic application from bench to bedside.

## Figures and Tables

**Table 1:** Pathogenic proteins contributing to EBV-associated NPC.

EBV target	Protumourigenic function	References
*Latent proteins*		

LMP1	Promoting interferon regulatory factor-7 (IRF-7) mediated angiogenesis and cell growth	[21]
	Inducing cancer stem-like cells in NPC	[22]
	Inducing tumour-promoting inflammation through NF-kB pathway	[23]
	Promoting expression of antiapoptotic proteins and inactivating proapoptotic proteins	[23]
	Stimulating cell growth by upregulating growth factor receptors (e.g., c-Met)	[24]

LMP2A	Inducing cancer stem-like properties by activating Hedgehog signalling pathway	[22]
	Promoting cancer cell migration and invasion that leads to metastasis	[25, 26]
	Inducing epithelial-mesenchymal transition (EMT) and enhancing side-population cells	[7]
	Activating PI3 K-AKT pathway to inhibit cellular differentiation and promote cancer cell survival	[27]
	Counteracting inhibitory and proapoptotic effects of TGF-B1 through PI3 K-AKT pathway	[28]
	Modulating mTOR pathway for cell survival and proliferation	[29]

EBNA1	Maintaining stable number of EBV genomes in infected cells	[5]
	Inducing genomic instability	[30]
	Reducing p53 levels and promoting cell survival	[31]
	Suppressing TGF-B1 signalling and promoting oncogenesis	[32]
	Inducing cellular DNA damage	[33]

*Lytic proteins*		

Zta	Induction of IL-8	[34]
BGLF5/DNase	Inducing genomic instability	[35]
BALF3	Inducing genomic instability	[36]
BARF	Increasing cell proliferation rate	[37]

**Table 2:** EBV targets for the development of NPC immunotherapy and therapeutic vaccine in the past 10 years.

CTL specificity	Prominent effects	Side effects	References
*EBV-specific cytotoxic T-lymphocytes (CTLs) immunotherapy*			

LMP1 & LMP2	Majority of pulmonary lesions were no longer evident in patients with recurrent NPC although primary tumour did not regress	Side effects such as fatigue, weakness, arthralgia, pain, haemoptysis, and epistaxis	[15]
	Highly efficient on patients with relapsed/refractory NPC (62% remains disease-free up to 75 months) particularly with locoregional disease	No long-term toxicity was reported	[51]
	6 out of 11 NPC patients, in whom conventional treatment has failed, showed either tumour regression or disease stabilization lasting more than 4 months	Four patients developed grade 3 neutropenia. Two patients suffered grade 2 thrombocytopenia. One patient suffered grade 2 anaemia. Mild toxic effects such as fatigue and nausea were observed in 6 patients	[52]

LMP1 & LMP2, EBNA1	Refractory NPC patients showed a median survival of 478 days, while patients with no or minimal residual disease remain alive	10 out of 30 patients suffered grade 1 adverse events; 6 out of 30 suffered grade 2 adverse events and 2 out of 30 suffered grade 3	[53]
	The median overall survival increased from 220 to 530 days compared with patients who did not receive the therapy	Few patients have been reported to have flu-like symptoms, malaise, dry cough, and low blood pressure	[54]

LMPs, EBNAs, BZLF1, BRKF1, BRMF1	Combination treatment of gemcitabine and carboplatin (GC) and CTL resulted in improved survival outcomes	Most of the reported side effects were grade 1. Mild toxic effects such as rash, fever, and fatigue were seen	[55]

LMP2, EBNAs, lytic proteins (BZLF1, BRLF1, BMLF1)	Patients with recurrent NPC produced higher number of autologous CTLs following CD45 mAbs	Grade 1 and 2 nonhematologic toxicities were observed among patients, including fever, abdominal pain, hypotension, and nausea. Transient neutropenia was also observed	[49]

*EBV-based therapeutic vaccine*			

Truncated LMP1 & full-length LMP2	Induced delayed type hypersensitivity (DTH) responses in 9 out of 12 patients	Mild nonhematological toxic effects such as fever, fatigue, and skin rash were observed in 9 patients. Total of 15 patients suffered grade 1/2 or 3 anaemia	[56]

LMP2 & EBNA1	Increased the T-cell responses in 15 of 18 patients	Reported negative reactions at injection sites. Flu-like symptoms, fatigue, arthralgia, myalgia, headache/dizziness, and hepatotoxicity were observed	[20]
	Increased immunity and induced differentiation and functional diversification of responsive T-cell populations	Grade 1 and 2 injection site reaction was observed in all participants. Nine patients experienced systemic toxicity	[19]
